# Differentiating Inhibitors of Closely Related Protein Kinases with Single- or Multi-Target Activity via Explainable Machine Learning and Feature Analysis

**DOI:** 10.3390/biom12040557

**Published:** 2022-04-08

**Authors:** Christian Feldmann, Jürgen Bajorath

**Affiliations:** Department of Life Science Informatics and Data Science, B-IT, LIMES Program Unit Chemical Biology and Medicinal Chemistry, Rheinische Friedrich-Wilhelms-Universität, Friedrich-Hirzebruch-Allee 5/6, D-53115 Bonn, Germany; cfeldmann@bit.uni-bonn.de

**Keywords:** protein kinases, human kinome, kinase inhibitors, single- and multi-target activity, explainable machine learning, structural features

## Abstract

Protein kinases are major drug targets. Most kinase inhibitors are directed against the adenosine triphosphate (ATP) cofactor binding site, which is largely conserved across the human kinome. Hence, such kinase inhibitors are often thought to be promiscuous. However, experimental evidence and activity data for publicly available kinase inhibitors indicate that this is not generally the case. We have investigated whether inhibitors of closely related human kinases with single- or multi-kinase activity can be differentiated on the basis of chemical structure. Therefore, a test system consisting of two distinct kinase triplets has been devised for which inhibitors with reported triple-kinase activities and corresponding single-kinase activities were assembled. Machine learning models derived on the basis of chemical structure distinguished between these multi- and single-kinase inhibitors with high accuracy. A model-independent explanatory approach was applied to identify structural features determining accurate predictions. For both kinase triplets, the analysis revealed decisive features contained in multi-kinase inhibitors. These features were found to be absent in corresponding single-kinase inhibitors, thus providing a rationale for successful machine learning. Mapping of features determining accurate predictions revealed that they formed coherent and chemically meaningful substructures that were characteristic of multi-kinase inhibitors compared with single-kinase inhibitors.

## 1. Introduction

In drug discovery, the ability of small molecules to interact with more than one protein in a well-defined manner provides the basis of polypharmacology; that is, the induction of the desired (or undesired) in vivo effects of drugs through the engagement of multiple targets [1,2,3,4,5]. Such multi-target activities of small molecules are a topic of intense investigation, from different perspectives [1,2,3,4,5,6,7,8,9]. Multi-target compounds (MT-CPDs) might be identified, for example, using profiling assays or proteomics techniques [10,11,12]. However, rationalizing multi-target activities of compounds (also referred to as promiscuity) at the molecular level of detail, distinguishing true activities from assay artifacts, and understanding how MT-CPDs might differ from single-target compounds (ST-CPDs) are far from being trivial tasks [13,14,15,16,17]. 

In addition to experimental methods, computational data analysis and predictive modeling are also applicable to aid in the analysis of the multi-target activities of small molecules [6,7,8,9]. For example, machine learning (ML) on the basis of chemical structure has been successfully used to systematically distinguish between MT-CPDs and corresponding ST-CPDs from medicinal chemistry or biological screening, thus providing evidence for the presence of structural features that differentiate MT- and ST-CPDs [18,19]. Furthermore, it has been shown through ML that structural features setting MT- and ST-CPDs apart strictly depend on the targets these compounds are active against and cannot be generalized [20]. 

Since ML model decisions are typically difficult to rationalize, the identification of structural features that determine accurate predictions requires the application of approaches for explaining ML [21,22,23,24,25]. Therefore, as a model-independent approach, the Shapley value concept [26] from game theory [27] was considered, which was originally developed to determine the contributions of individual players to the performance of a team [26]. In ML model interpretation, this concept can be applied to quantify the contributions of individual features to the prediction of a test instance. To ensure computational feasibility for large feature sets typically used in ML, a local interpretation model can be derived that approximates a complex ML model in a given region of feature space for individual predictions. This local approach is termed Shapley Additive exPlanations (SHAP) [28] and has been adapted for explaining compound activity predictions [29]. SHAP values quantify the contributions of features that are present or absent in a compound to a prediction, and the sum of all feature contributions yields the probability of this prediction. This local approach is applicable to any ML method to approximate SHAP values. Moreover, for decision tree-based methods, an algorithm has been introduced for the calculation of exact SHAP values [30].

Using the tree-based SHAP approach, we previously attempted to identify the structural features that determined the accurate prediction of compounds active against given target pairs (dual-target compounds, DT-CPDs) vs. corresponding ST-CPDs [31]. Targets forming investigated pairs were structurally and functionally unrelated. SHAP feature importance analysis identified structural motifs in DT-CPDs that differentiated them from ST-CPDs and that were implicated in polypharmacology [31]. 

In this work, we aimed to differentiate compounds with reported activity against single or multiple protein kinases, representing a scenario completely distinct from investigating compounds that are active against unrelated targets. Kinases represent a major class of drug targets [32] and the efficacy of kinase inhibitors often relies on polypharmacology, especially in oncology [32,33]. Most currently available kinase inhibitors target the ATP cofactor binding site that is largely conserved across the human kinome [34,35]. Thus, ATP site-directed kinase inhibitors are expected to be promiscuous [33,34], although the results of experimental kinase profiling campaigns [11,12] and the analysis of publicly available compound activity data [7,35] do not generally support this notion. Many ATP site-directed kinase inhibitors are only annotated with a single kinase [35], and it often remains unclear to what extent differences in the experimental test frequency of kinase inhibitors are responsible for the presence or absence of multi-kinase annotations. 

In order to investigate compounds with different activity against closely related targets, we assembled inhibitors with reported activity against triplets or in part very closely related kinases and other inhibitors only reported to be active against one of these kinases. First, we addressed the question of whether these corresponding single- and triple-kinase inhibitors (ST- and MT-CPDs, respectively) can be distinguished using ML models exclusively derived on the basis of chemical structure. Second, we attempted to rationalize the results of these predictions using the SHAP formalism and identified structural features decisive for prediction outcomes. Third, the findings were interpreted from a chemical perspective. 

## 2. Materials and Methods 

### 2.1. Compounds and Activity Data

Compounds annotated with standard potency measurements (K_i_, IC_50_, or K_d_) and an exact potency value (“=”) of at least 10 µM against human kinases were extracted (and recorded as negative decadic logarithmic values) from ChEMBL (version 29) [36]. Only direct interactions (target relationship type: “D”) with wild-type proteins at the highest confidence level (target confidence score: 9) were considered, omitting measurements flagged as “potential author error” or “potential transcription error.” Compounds with a mass of 1000 Da or more and potential assay interference compounds were removed using public tools and filters [16,37,38]. 

### 2.2. Target Selection

A search was carried out for kinase triplets including at least two closely related kinases from the same family for which sufficient numbers of MT- and corresponding ST-CPDs for meaningful ML and feature analysis were available (see Results and Discussion). Based on available inhibitors with high-confidence activity data and the applied selection criteria, two kinase triplets were prioritized, as reported in Table 1. In addition to focal adhesion kinase 1 (FAK1), triplet 1 contained two Januskinases, that is, Janus kinase 2 (JAK2) and 3 (JAK3). Triplet 2 comprised three closely related dual specificity tyrosine-phosphorylation-regulated kinases (DYRK1A, DYRK1B, and DYRK2). For triplet 1, larger numbers of compounds were available than for triplet 2. A generally limiting factor for triplet assembly was the limited availability of sufficient numbers of MT-CPDs with high-confidence activity data, which were essential for the analysis. Nine of the ST-CPDs for FAK1 (triplet 1) were designated allosteric compounds. All other inhibitors for both triplets, including all MT-CPDs, were ATP-competitive.

### 2.3. Molecular Representation

As a molecular representation, atom environments were selected as preferred topological features [20,39]. The RDKit [37] implementation of the Morgan fingerprint corresponding to the extended connectivity fingerprint [39] was utilized to generate hash values of molecule-specific layered atom environments (up to a bond radius of 2, corresponding to a bond diameter of 4) for each atom in a compound. Obtained feature hashes were assigned to unique positions in the final feature vector to avoid bit collisions, thereby ensuring the interpretability of calculated feature importance values.

### 2.4. Machine Learning

Compounds were classified using a balanced random forest (BRF) model consisting of an ensemble of decision trees [40,41]. For each tree, a unique bootstrap sample of the training set was drawn and subsequently balanced by randomly under-sampling the majority class. This approach allowed us to utilize the majority of training data under the condition of class balance; an important criterion, given the presence of significantly different numbers of MT- and corresponding ST-CPDs. Predicted probabilities for multi-target activity were calculated as the mean probability over individual trees, which estimated the class probability as the fraction of samples of the given class in the final leaf node. Hyperparameters such as number of decision trees (“n_estimators”: 25, 50, 100, 200, 400), minimal number of samples for a split (“min_samples_split”: 2, 3, 5, 10), and minimum number of samples for a leaf node (“min_samples_leaf”: 1, 2, 5, 10) were assessed via internal 10-fold shuffle-split cross validation on the training set. The final MT- vs. ST-CPD classifier was trained with the best-performing hyperparameter combination using the complete training set, representing a random sample of 75% of the compounds. Model performances were assessed using a balanced sample of the remaining 25% of MT- and ST-CPDs as test instances over 10 individual trials. As performance measures, balanced accuracy (BA) [42], F1-score (F1) [43], precision, recall, and Matthews correlation coefficient (MCC) [44] values were calculated. These performance measures are defined as follows:

BA = 1/2(TPR + TNR)

F1 = TP/(TP + 1/2(FP + FN))

Precision = TP/(TP + FP)

Recall = TP/(TP + FN)

MCC = (TP × TN-FP × FN)/√((TP + FP)(TP + FN)(TN + FP)(TN + FN))

TP, TN, FP, and FN stand for true positives, true negatives, false positives, and false negatives, respectively.

### 2.5. SHAP Analysis and Feature Extraction

The use of BRF models enabled the accurate calculation of SHAP feature importance values for individual predictions using the TreeExplainer algorithm with tree-path dependent feature perturbations [30]. SHAP theory is provided in the Supplementary Methods, and cumulative SHAP feature contributions yielding a class label probability are illustrated in Supplementary Figure S1.

A feature extraction scheme was devised for correctly predicting instances taking into account that SHAP feature importance values might differ from test instance to test instance [31]. This feature extraction scheme bridges instance predictions and SHAP feature importance across all test instances, as follows:

(i) For each correctly predicted MT-CPD, the top-ranked N features with the highest SHAP values were pre-selected and these features were pooled.
(ii) The pool of the top-ranked N features was re-ranked by the feature frequency of occurrence in correctly predicted MT-CPDs, and the top M most frequent features were selected.

For the calculations reported herein, N = 5 and M = 10 settings were consistently applied.

## 3. Results and Discussion

### 3.1. Study Design

The newly generated kinase triplet test system with available MT- and ST-CPDs, as described below, enabled us to first address the key question if multi-kinase inhibitors could be systematically distinguished from single-kinase inhibitors by ML on the basis of chemical structure information. Given the frequent assumption that kinase inhibitors tend to be promiscuous, this was not necessarily likely. If accurate classification of MT- vs. ST-CPDs is possible, however, then structural features detectable by ML must exist that differentiate MT- and ST-CPDs and hence determine accurate predictions. If so, the second step of the analysis then aims at identifying these features via an independent explanatory approach (SHAP). Whether or not features determining algorithmic predictions might be chemically relevant and explainable in chemical terms was another open question. Therefore, in the third step, we aimed at rationalizing distinguishing features (provided they were identified) from a chemical perspective. Hence, the analysis was designed to identify the structural features driving the correct prediction of ST- and/or MT-CPDs, which might also be implicated in kinase selectivity or promiscuity, respectively.

### 3.2. Systematic Analysis of Kinase Triplets

**Figure 1:**
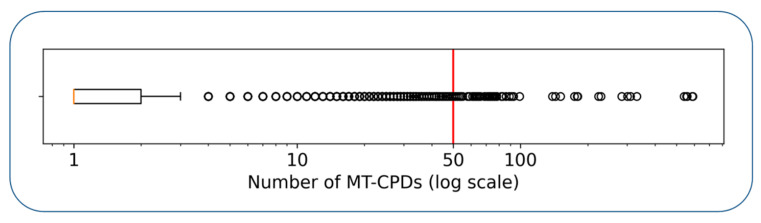
MT-CPDs for systematically explored kinase triplets. The boxplot shows the number of MT-CPDs (on a logarithmic scale) for all possible kinase triplets with at least one available MT-CPD. The left and right boundaries of the box indicate the upper and lower quartile of the distribution, while the vertical orange line represents the median value. The whisker shows the maximum of the distribution. Values exceeding the maximum are statistical outliers and depicted as circles.

The 489 human kinases with available active compounds were systematically organized into triplets, yielding nearly 20 million (19,368,964) unique combinations. Of these possible combinations, 6,132,688 were found to share at least one inhibitor. Figure 1 reports the distribution of the number of MT-CPDs for these triplets, revealing that ~75% of all triplets had no more than two MT-CPDs and only 64 triplets had at least 50 MT-CPDs. Hence, confirmed MT-CPDs were generally rare. 

As a minimal amount of negative data, triplets were required to have at least 17 ST-CPDs for each kinase, reducing the number of triplets to 57. For 35 of these triplets, a mean balanced accuracy of BRF models greater than 80% was observed (see below) and SHAP calculations prioritized features determining the predictions. For the 35 triplets, the number of MT-CPDs ranged from 51 to 310. As representative triplets for subsequent analysis, triplet 1 with very high prediction accuracy and a large number of available MT-CPDs, and triplet 2 with lower prediction accuracy and a smaller number of MT-CPDs were chosen. In both instances, features decisive for the predictions were clearly interpretable in chemical terms (which is not necessarily the case in ML).

### 3.3. Compound Classification

**Figure 2:**
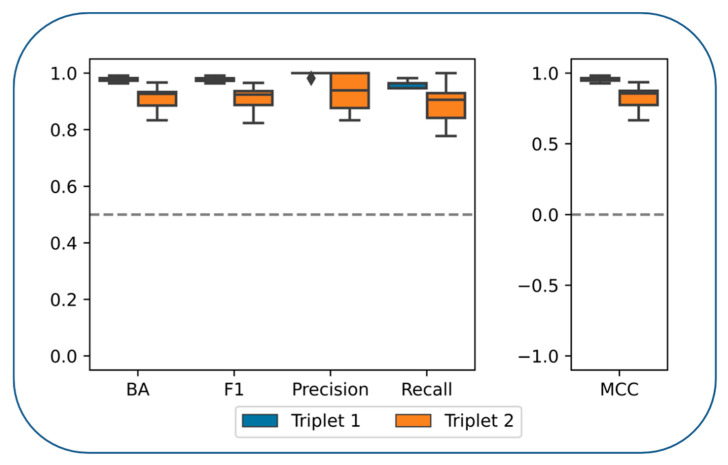
Performances of the balanced random forest classifier. Boxplots represent distributions of balanced accuracy, F1 score, precision, recall and MCC over 10 individual trials for triplet 1 (blue) and triplet 2 (orange). In a boxplot, the horizontal line indicates the median value of the distribution and the upper and lower boundaries of the box indicate the upper and lower quartile, respectively. In addition, whiskers represent the maximum and minimum values of the distribution, and statistical outliers are depicted as diamonds.

Figure 2 summarizes the performance of our BRF models. For both kinase triplets, MT- and ST-CPDs were distinguished with surprisingly high accuracy, as determined on the basis of different performance measures. The calculations were generally stable, as reflected by the narrow distributions of the results obtained over independent trials. For triplet 2, prediction accuracy was consistently above 80%. However, for triplet 1, the predictions were nearly perfect, with values of all performance measures approaching 1.0 (the trial set-ups and results were thoroughly re-examined, excluding the presence of artifacts for triplet 1). Taken together, these findings provided evidence for the presence of distinguishing structural features and an unexpectedly solid foundation for subsequent feature analysis.

### 3.4. Representation Features Determining Predictions

On the basis of the BFR results, SHAP analysis was carried out for each correctly predicted MT- and ST-CPD, prioritized features were extracted (see Materials and Methods), and their contributions to accurate predictions were quantified. The analysis was carried out for all representation (fingerprint) features that were present in test compounds as well as for features that were absent, thus comprehensively searching for features determining correct predictions. 

**Figure 3:**
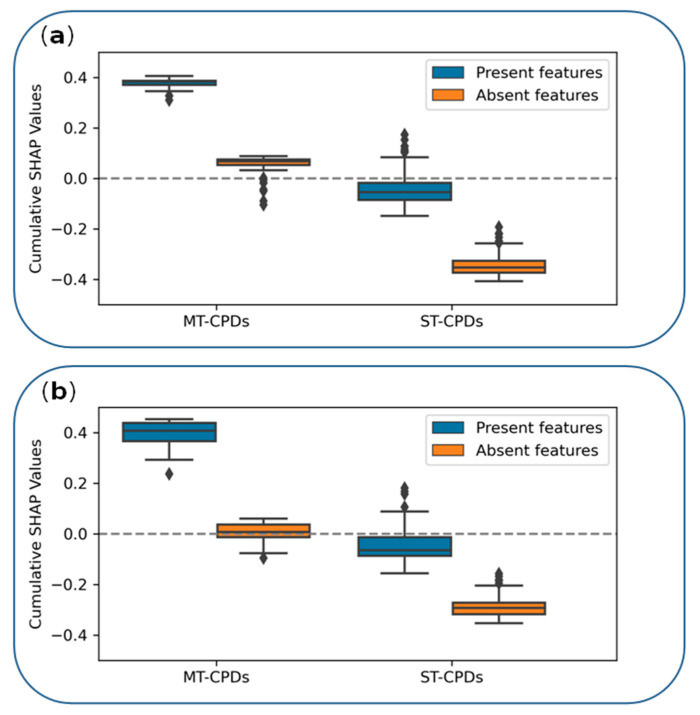
Feature contributions. Boxplots report the distributions of cumulative SHAP values of features present (blue) or absent (orange) in correctly predicted MT- or ST-CPDs. (**a**,**b**) show the results for triplet 1 and triplet 2, respectively.

Figure 3 shows the results of SHAP feature importance analysis. For both triplets 1 and 2, a clear and consistent picture emerged from the analysis. Accurate predictions of MT-CPDs were determined by the features that were present in these compounds. These features made large positive contributions, whereas features absent in MT-CPDs made only small positive or negative contributions to the predictions (which essentially canceled out). By contrast, correct predictions of ST-CPDs were largely determined by features that were absent in these compounds (but present in MT-CPDs). In this case, present features only made small supporting contributions (i.e., negative in the case of the ST-CPD class) or opposing (positive) contributions. These observations paralleled our previous findings for MT- and ST-CPDs with activity against pairs of unrelated targets [31]. Thus, for kinase inhibitors, ML successfully distinguished between MT- and ST-CPDs on the basis of structural features that were unique to MT-CPDs. 

### 3.5. Feature Mapping and Rationalization

**Figure 4:**
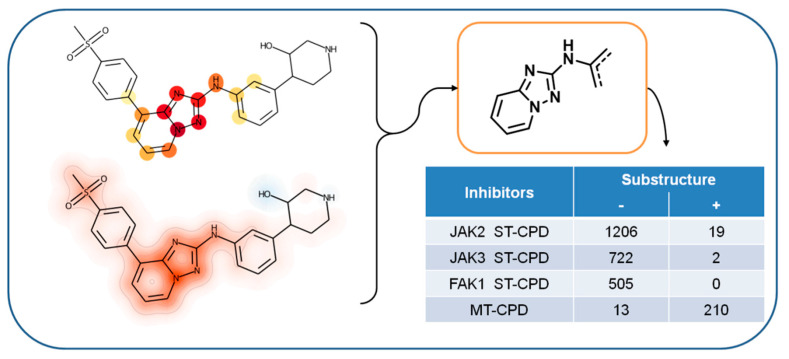
Feature mapping for MT-CPDs of triplet 1. Extracted features are mapped on exemplary correctly predicted MT-CPDs (upper left) on a per-atom basis. The continuous atom color code indicates the number of extracted features per atom, ranging from 1 (yellow) to 10 (dark red). In addition, SHAP values for all present features were mapped to the corresponding atoms and shown in a heat-map format (lower left). Both representations identify the [1,2,4]triazolo[1,5-a]pyridine substructure (upper right) as the center of positive feature contributions to the correct prediction of this MT-CPD and others containing this substructure. The presence (+) or absence (−) of the substructure in ST- and MT-CPDs of triplet 1 is reported in the table insert (lower right).

**Figure 5:**
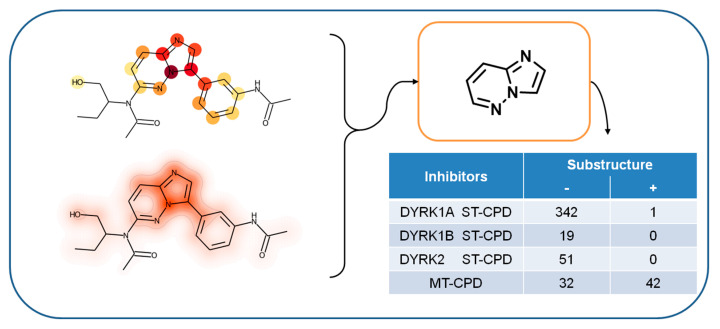
Feature mapping for MT-CPDs of triplet 2. The presentation is according to Figure 4. In the molecular representation on the upper left, the continuous atom color code indicates the number of extracted features per atom, ranging from 1 (yellow) to 11 (brown). Both molecular representations identify the imidazo(1,2-b)pyridazine substructure (upper right) as the center of positive feature contributions to the correct prediction of this MT-CPD and others containing this substructure. The presence (+) or absence (−) of the substructure in ST- and MT-CPDs of triplet 1 is reported in the table insert (lower right).

After extracting and mapping individual features determining the accurate prediction of MT-CPDs, we annotated atoms of MT-CPDs with SHAP values from all features present in the respective compounds (including extracted features) for further analysis. Using layered atom environments (consisting of atom sets) as representation features made it possible to unambiguously map these contributions on a per-atom basis. For MT-CPDs from both triplets 1 and 2, highlighted regions were not evenly distributed over the compound structure, but delineated a coherent substructure, as shown in Figure 4 and Figure 5, respectively. Hence, the mapping of structural features determining the accurate prediction of MT-CPDs identified a well-defined structural motif. In both cases, this structural motif was predominantly formed by extracted (prioritized) features. In ST-CPDs, similar structural features influencing the predictions were not detected, as discussed above. For triplet 1, the delineated substructure was a [1,2,4]triazolo[1,5-a]pyridine, as depicted in Figure 4.

The [1,2,4]triazolo[1,5-a]pyridine substructure was present in 210 of 223 MT-CPDs, but in only 19 of 1206 JAK2, 2 of 722 JAK3 ST-CPDs and none of the 505 FAK1 ST-CPDs. Thus, the substructure was characteristic of triplet 1 MT-CPDs. This finding also explained the decisive role of extracted features defining this substructure for accurate predictions. All 210 MT-CPDs containing this substructure originated from a single patent source establishing triple-kinase activity [45]. It follows that the 21 ST-CPDs containing the [1,2,4]triazolo[1,5-a]pyridine might also have triple-kinase activity. On the other hand, since only 21 of 2433 ST-CPDs contained this substructure, it is very likely to represent a chemical signature of MT-CPDs.

For triplet 2, the substructure in MT-CPDs identified by feature mapping was imidazo(1,2-b)pyridazine, as depicted in Figure 5. In this case, features present in MT-CPDs were more widely distributed across the compound structure, while the imidazo(1,2-b)pyridazine was highlighted by mapped SHAP values. This was consistent with the observation that this substructure was contained in 42 of 74 MT-CPDs (which also originated from a single study establishing triple-kinase activity [46]). For the remaining 32 MT-CPDs, extracted features did not delineate another well-defined structural motif. However, the imidazo(1,2-b)pyridazine substructure was also characteristic of the large subset of 42 MT-CPDs because it did not occur in any DYRK1B or DYRK2 ST-CPD, and only in 1 of 342 DYRK1A ST-CPDs.

**Figure 6:**
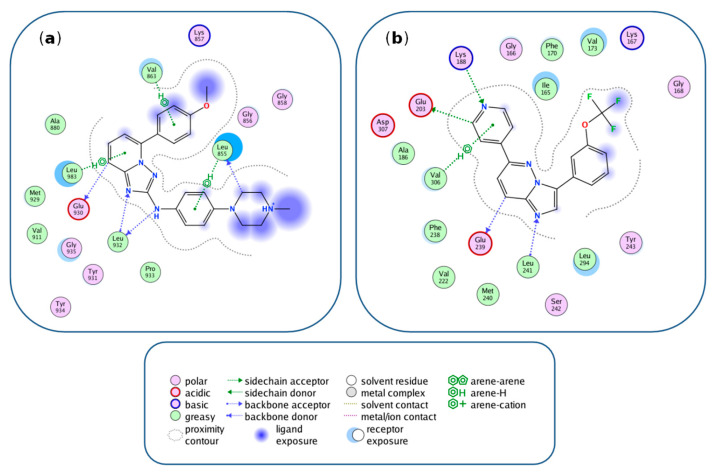
Kinase interactions with inhibitors containing characteristic substructures. Shown are kinase–inhibitor interaction diagrams derived from X-ray structures of kinase–inhibitor complexes using the Molecular Operating Environment (MOE, Chemical Computing Group, Inc., Montreal, QC, Canada). (**a**) shows a crystallographic inhibitor containing the [1,2,4]triazolo[1,5-a]pyridine substructure characteristic of MT-CPDs of triplet 1 in complex with JAK2 (PDB ID: 4JIA) and (**b**) an inhibitor with the imidazo(1,2-b)pyridazine substructure of MT-CPDs of triplet 2 in complex with DYRK1A (PDB ID: 6S11, Chain: A). At the bottom, different types of interactions accounted for in the diagram are specified. These interaction types were automatically classified using MOE. We note that under physiological conditions, interactions between the charged glutamic acid residue and aromatic rings should best be perceived as anionic–π_aromatic_ interactions.

For both characteristic substructures, we also found an X-ray structure of a complex formed between an inhibitor containing the substructure and a kinase from triple 1 and triple 2, respectively. Figure 6 shows kinase–inhibitor interaction diagrams computed from these X-ray structures. The [1,2,4]triazolo[1,5-a]pyridine moiety in the inhibitor in Figure 6a was involved in multiple interactions with JAK2, forming the center of interactions for this inhibitor in the ATP binding site of the kinase. The imidazo(1,2-b)pyridazine moiety of the inhibitor in Figure 6b also formed interactions with DYRKA1 (but was not an interaction hot spot). Both characteristic substructures were located in the same region of the ATP site (essentially mimicking the adenosine moiety in ATP) and also interacted with residues conserved in the ATP site of other kinases, consistent with the multi-kinase activity of inhibitors containing these substructures.

## 4. Conclusions

In this work, we attempted to differentiate inhibitors with triple-kinase or corresponding single-kinase activity by ML on the basis of chemical structure, identify features determining accurate predictions, and interpret key features in chemical terms. Exploring the molecular origins of the varying promiscuity of ATP site-directed kinase inhibitors continues to be a topic of intense investigation in medicinal chemistry. For our analysis, we generated a test system consisting of kinase triplets for which sufficient numbers of MT- and ST-CPDs with high-confidence activity data were available to enable meaningful ML. Moreover, we specifically aimed to investigate closely related kinases from the same family most likely to have similar compound-binding characteristics. MT- and ST-CPDs from kinase triplet 1 and 2 were differentiated with high accuracy using ML models, providing evidence for the presence of distinguishing structural features. SHAP analysis then identified features determining the predictions. An important finding shows that accurate predictions resulted from features that were present in MT- but absent in ST-CPDs. These features were found to be chemically sensible, forming coherent substructures that were characteristic of MT-CPDs. ML prediction accuracy was nearly perfect for kinase triplet 1 and in this case, the characteristic substructure was present in 94% of all MT- and absent in 99% of all ST-CPDs, thus reflecting the high consistency of ML results and feature analysis. Taken together, the findings reported herein have methodological implications as well as implications for kinase inhibitor research. From a methodological point of view, our results clearly support the utility of explainable ML to rationalize predictions from a chemical or biological perspective and reveal structural information important for drug discovery and design, as exemplified by the identification of substructures characteristic of MT-CPDs. However, despite the consistency of the obtained results, they are difficult to generalize for kinase inhibitor research. ML and feature analysis, at least in part, depend on the composition of the investigated data sets and care must be taken not to over-interpret the findings. For example, while we can conclude with certainty from our analysis that the [1,2,4]triazolo[1,5-a]pyridine and imidazo(1,2-b)pyridazine moieties identified herein are signatures of multi-kinase activity, we cannot conclude that the designated ST-CPDs assembled on the basis of currently available activity data are kinase-selective. Here, varying test frequencies among kinase inhibitors might come into play that are for the most part unknown for compounds collected from different sources and thus cannot be considered in the computational analysis. On the other hand, it makes perfect sense that designated ST-CPDs do not share characteristic structural features determining their prediction. The presence of such features in ST-CPDs would principally not be consistent with kinase selectivity, while the absence of shared features supports differences between these compounds. Clearly, such considerations are important for putting the results into perspective. However, explainable ML as presented herein yields, at the very least, experimentally testable hypotheses for distinguishing between inhibitors with single- and multi-kinase activity and for exploring further structural features implicated in promiscuity vs. selectivity.

## Figures and Tables

**Table 1:** Kinase triplets and compound statistics.

	Annotation	Number of Inhibitors
**Triplet 1**	(Triple-target) MT-CPDs	223
	ST-CPDs Tyrosine-protein kinase JAK2	1225
	Tyrosine-protein kinase JAK3	724
	Adhesion kinase 1	505
		
**Triplet 2**	(Triple-target) MT-CPDs	74
	ST-CPDs Dual specificity tyrosine-phosphorylation-regulated kinase 1A	343
	Dual specificity tyrosine-phosphorylation-regulated kinase 1B	19
	Dual specificity tyrosine-phosphorylation-regulated kinase 2	51

## Data Availability

Compounds and activity data were obtained from the publicly available ChEMBL database (https://www.ebi.ac.uk/chembl/ (Accessed on 10 February 2022)).

## Supplementary Materials

The following supporting information can be downloaded at: https://www.mdpi.com/article/10.3390/biom12040557/s1, Supplementary Methods [47]: SHAP theory; Figure S1: SHAP feature importance.
