# Supplementary material for: Differentiating Inhibitors of Closely Related Protein Kinases with Single- or Multi-Target Activity via Explainable Machine Learning and Feature Analysis
Source: Biomolecules. 2022 Apr 8;12(4):557. doi: 10.3390/biom12040557 (PMC9032434; doi:10.3390/biom12040557)
Supplement: Supplementary file 1 [file biomolecules-12-00557-s001.zip › biomolecules-1665307-supplementary.pdf]

Supplementary Materials

Differentiating Inhibitors of Closely Related Protein Kinases with Single- or Multi-Target Activity via Explainable Machine Learning and Feature Analysis

Christian Feldmann and Jürgen Bajorath

Department of Life Science Informatics and Data Science, B-IT, LIMES Program Unit Chemical Biology and Medicinal Chemistry, Rheinische Friedrich-Wilhelms-Universität, Friedrich-Hirzebruch-Allee 6, D-53115 Bonn, Germany;

*Correspondence: bajorath@bit.uni-bonn.de; Tel.: +49-228-7369-100.

Supplementary Methods, Supplementary Figure S1

Supplementary Methods

SHAP theory

Shapley values were originally introduced to estimate the importance of contributions of an individual player in a collaborative team. Therefore, the total gain among players is distributed depending on the relative importance of their contributions to the final outcome of a game. Shapley values represent a unique reward for each participating player obtained through the assessment of contributions resulting from all possible orderings of players and their contributions.

The Shapley value concept can be applied to explain individual predictions of ML models by applying the following analogies [24]:

(1) The *game* a team engages in can be perceived as a *prediction task for a single instance* (e.g., a compound). The merit for this task is given by the difference between its prediction and the average prediction of all instances.

(2) The *players* participating in the game are *features values of the instance* that cooperate (act jointly) to achieve the merit for the given prediction. The resulting Shapley value of a given feature is then obtained as the average contribution of a feature over all possible feature combinations.

Accordingly, Shapley values account for the partition of contributions over individual features comprising a feature vector or set (such as a molecular representation). A key aspect of the

Shapley value concept is that *not only the contribution of feature presence to a given prediction can be quantified, but also the contribution of feature absence* [24,31].

For feature sets of increasing size, systematic calculations of Shapley values on the basis of all possible feature combinations become computationally demanding or infeasible. Therefore, for ML, a *locally interpretable explanatory model* has been introduced, which approximates Shapely values heuristically. This local methodology is termed *Shapley Additive exPlanations* (SHAP) [28] and can be perceived as an extension of the *Local Interpretable Model-agnostic Explanations* (LIME) approach [47].

The principal goal of an explanation model g is to locally approximate and thus simplify a complex model f that is difficult to understand. Additive feature attribution methods generate an explanation model via a linear function of binary variables, given by *Equation 1*:

$$g(x') = \phi_0 + \sum_{i=1}^{M} \phi_i x'_i \quad (1)$$

where $x' \in \{0,1\}^M$, M is the number of input features, and $\phi_i \in \mathbb{R}$. The presence or absence of a feature value impacting the prediction yields a feature contribution (ϕ_i). Accordingly, a weight must be assigned to each variable for which the LIME methodology [4] can be applied and further extended. LIME generates the explanation ξ of an instance x according to *Equation 2:*

$$\xi(x) = \underset{g \in G}{\operatorname{argmin}} \mathcal{L}(f, g, \pi_x) + \Omega(g) \quad (2)$$

where G is a class of interpretable (linear) models, $\mathcal{L}$ is the loss function to minimize, π_x the proximity measure between an instance z and x (kernel defining locality), and $\Omega(g)$ an optional regularization term to limit model complexity.

For the explanation of a given test instance x, the following procedure is applied:

(i) Artificial samples are obtained by permuting features of the test instance x.

(ii) These samples are weighted by the value of a kernel calculated for them and x.

(iii) A model g is trained to predict *f(x)* with coefficients estimating feature importance.

Accordingly, LIME builds a linear model g in a feature region proximal to the test instance, with ML model f typically being non-linear. The LIME approach provides the basis for the development of the kernel SHAP methodology, as explained in the following.

Shapley values account for the distribution of feature contributions to a model's prediction for a given test instance. To determine the contribution of a feature *i*, all operations by which a feature might be added to the set ($N!$) and a summation over all possible sets (S) must be carried out. For any feature sequence, the marginal contribution by adding feature i is given by $[f(S \cup \{i\}) - f(S)]$. The resulting quantity is weighted by the number of combinations available to form the set prior to addition of feature I, i.e., ($|S|!$), and the order in which remaining features might be added, i.e., ($(|N| - |S| - 1)!$). Hence, the importance of a given feature *i* is defined by *Equation 3:*

$$\phi_i = \frac{1}{N!} \sum_{S \subseteq N \setminus \{i\}} |S|! \, (|N| - |S| - 1)! \, [f(S \cup \{i\}) - f(S)] \quad (3)$$

Shapley values thus represent a unique way of dividing a model's output among feature contributions satisfying three axioms: *local accuracy* (or additivity), *consistency* (or symmetry), and *nonexistence* (or null effect).

Additive feature attribution methods typically do not consider two properties that are of high relevance for assessing feature importance including *local accuracy* and *consistency*. The SHAP formalism was devised to take these axiomatic properties into account [28]. The property *local accuracy* ensures that the sum of individual feature attributions is equal to the original prediction because SHAP allocates the model prediction across contributing features. Furthermore, *consistency* ensures that feature importance correctly accounts for different models on a relative scale. Hence, if a change in a feature value has larger impact on model A than model B, feature importance should be larger in A. These properties can be accounted for by representing feature importance as SHAP values [28].

A weighting procedure for artificial samples is a key aspect for connecting Shapley values to the LIME approach. In LIME, heuristic choices are made to select $\mathcal{L}$, $\Omega(g)$, and π_x. By contrast, the SHAP method introduces a special kernel function that is related to the Shapley value definition, assuming that feature weights follow the two axioms of interpretability. Specifically, SHAP uses the following procedure for interpreting an instance x:

(i) Training data are organized by k-means clustering and the k samples are weighted by the number of training instances they represent. These samples constitute a background data set of given feature values.

(ii) Artificial samples are obtained by replacing features of the test instance x with the values from the background data set.

(iii) These artificial samples are weighted by the value of the SHAP kernel calculated for each and x.

(iv) A weighted linear regression model g is trained to predict *f(x)*. The model coefficients are Shapley values corresponding to feature importance estimates.

Sampling all possible feature subsets is avoided through permutation of the feature vector by setting features on and off. A feature is assigned a large weight if its replacement with an artificial value leads to a significant change in model output. Weights of artificial samples are determined as the number of feature addition sequences of a given subset by the SHAP kernel. Coefficients from local linear regression provide feature weights as Shapley values, which indicate how important a feature is for a given prediction including the direction (sign) of feature influence. The expected explanatory value is calculated as the mean of the model output probability (or numerical value) over training set instances. For a given instance, the model output is then calculated as the sum of the expected (base) value and all SHAP feature values.

For an individual instance (compound), SHAP calculations yield quantitative feature contributions that support (positive value) or oppose (negative value) a given prediction. The sum of positive and negative contributions including the base value of the model (expected value, obtained as the mean feature importance value of training instances) results in a class label probability. Depending on the compound, different numbers of features might make positive or

negative contributions of varying magnitude, as accounted for by SVs. Importantly, SHAP analysis also quantifies contributions of features that are absent in a test instance. This ability is of critical relevance because the absence of specific features might be responsible for a given prediction just as much as the presence of another.

The SHAP theory section was in part adapted from [24] (our open access publication).

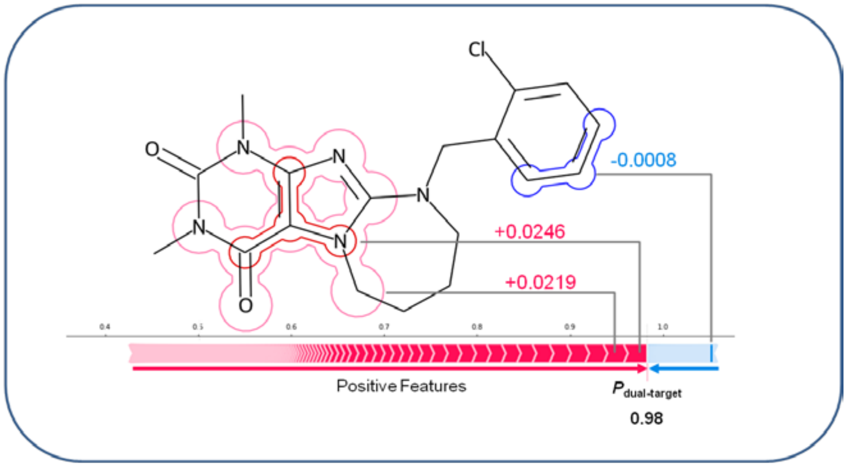

Supplementary Figure S1. SHAP analysis. For an exemplary compound, positive (red) and negative (blue) SHAP feature contributions yield a probability *P* of multi-target activity. In this case, contributions from all but one feature present in the compound are positive. The sum of the base value of the classifier (0.5) and all feature importance values results in a probability of multi-target activity of 0.98. The figure was adopted from [31].
